# Boron Oxide Enhancing Stability of MoS_2_ Anode Materials for Lithium-Ion Batteries

**DOI:** 10.3390/ma15062034

**Published:** 2022-03-10

**Authors:** Thang Phan Nguyen, Il Tae Kim

**Affiliations:** Department of Chemical and Biological Engineering, Gachon University, Seongnam 13120, Gyeonggi, Korea; phanthang87@gmail.com

**Keywords:** MoS_2_, B_2_O_3_, chemical exfoliation method, sintering techniques, lithium-ion batteries

## Abstract

Molybdenum disulfide (MoS_2_) is the most well-known transition metal chalcogenide for lithium storage applications because of its simple preparation process, superior optical, physical, and electrical properties, and high stability. However, recent research has shown that bare MoS_2_ nanosheet (NS) can be reformed to the bulk structure, and sulfur atoms can be dissolved in electrolytes or form polymeric structures, thereby preventing lithium insertion/desertion and reducing cycling performance. To enhance the electrochemical performance of the MoS_2_ NSs, B_2_O_3_ nanoparticles were decorated on the surface of MoS_2_ NSs via a sintering technique. The structure of B_2_O_3_ decorated MoS_2_ changed slightly with the formation of a lattice spacing of ~7.37 Å. The characterization of materials confirmed the formation of B_2_O_3_ crystals at 30% weight percentage of H_3_BO_3_ starting materials. In particular, the MoS_2__B3 sample showed a stable capacity of ~500 mAh·g^−1^ after the first cycle. The cycling test delivered a high reversible specific capacity of ~82% of the second cycle after 100 cycles. Furthermore, the rate performance also showed a remarkable recovery capacity of ~98%. These results suggest that the use of B_2_O_3_ decorations could be a viable method for improving the stability of anode materials in lithium storage applications.

## 1. Introduction

Low-dimensional layered structures of transition metal chalcogenides (TMCs) have attracted increased attention because of their superior properties, such as high conductivity, high stability, easy processing, and easy computing, in two-dimensional (2D) structures [1,2,3,4,5,6]. Therefore, various research has been undertaken to utilize TMCs in applications that traditionally used graphene materials [7,8,9,10]. Among them, MoS_2_ is the most well-known TMC material. MoS_2_ nanosheets (NSs) can be easily obtained through either top-down approaches, such as scotch tape, sonication, and chemical exfoliation, or bottom-up approaches, such as hydrothermal, chemical vapor deposition, and microwave-assisted methods. 2D MoS_2_ NSs possess high conductivity, flexibility, and a large surface area, thereby making them potential candidates for anode materials in lithium storage applications. The MoS_2_ NSs have a theoretical capacity of ~670 mAh·g^−1^, which is twice that of graphite (~372 mAh∙g^−1^) [11]. However, previous reports have shown that the MoS_2_ NS anodes undergo fast degradation due to the dissolution of sulfur atoms and dislocation of MoS_2_ nanosheets during the cycling process [12,13]. Moreover, the conversion reaction of MoS_2_ to form Li_2_S, the solid electrolyte interface (SEI) layer, and the degradation of the electrolyte resulted in the formation of a gel-like polymeric layer, which led to fast capacity fading [14]. Many attempts have been made to enhance the stability of MoS_2_ NSs based on the use of graphene/carbon nanotube (CNT)/carbon cloth as skeletons, carbon coating layers, or the addition of foreign materials (such as TiO_2_, MnO, Ag, and Sn) to prevent the restacking of MoS_2_ and co-contribute to the electrochemical conversion reaction with lithium [11,14,15,16,17,18,19,20]. For example, Kong et al. demonstrated that MoS_2_ nanoplates, with coverage of rolled-up graphene layers, form a core–shell MoS_2_@graphitic nanotube, which showed a high rate performance and high capacity without using a binder [21]. Yoo et al. used CNTs as skeletons to grow MoS_2_ via microwave irradiation [22]. The cylindrical-structured MoS_2_ on CNTs exhibited advantageous electrochemical properties, such as high rate and high stability, as anode materials in lithium-ion batteries (LIBs). Ren et al. combined both graphene and CNTs as a frame structure for the decoration of MoS_2_ nanoparticles (NPs), which delivered a high reversible capacity of ~600 mAh∙g^−1^ for 200 cycles [23]. Qu et al. decorated Fe_2_O_3_ NPs on MoS_2_ NSs via a hydrothermal method and sintering process, in which the anodes exhibited high-rate performances and a high reversible capacity of ~900 mAh∙g^−1^ [24]. Zhao et al. prepared the composition MoO_3_/MoS_2_, which has core–sheath structure, via a sulfurization technique [25]. The MoO_3_/MoS_2_ core–sheath anodes exhibited a negative fading phenomenon and achieved a capacity of ~1500 mAh∙g^−1^ after 150 cycles. Even though many attempts on improving the electrochemical performance of MoS_2_ NS have been made, the mechanisms are still not clearly revealed and further improvement in stability is still needed to meet the requirements of practical applications.

Recently, lithium nickel cobalt manganese oxide (NMC) cathode materials have been effectively enhanced their stability performance by using boron compounds, such as cobalt boride (Co_x_B) and B_2_O_3_, for surface modifications [26,27,28]. Yoon et al. revealed that Co_x_B metallic glass in Ni-rich NMC can effectively enhance the stability of cathode materials via reactive wetting [26]. Li et al. utilized B_2_O_3_ as a surface-modification material to enhance the performance of the NMC111 cathode [27]. The use of B_2_O_3_ also resulted in graphene combined with a MoS_2_ hierarchical structure, which improved the photo/electro properties of the graphene/MoS_2_ composition for bio applications [29]. Riyanto et al. reported that a boron-doped graphene quantum structure with MoS_2_ could deliver a high capacity of ~1000 mAh∙g^−1^ [30]. However, the effect of B_2_O_3_ in lithium-ion batteries has not been investigated. B_2_O_3_ is a low-cost material with low environmental pollution and easy processing, and it plays an important role in many applications such as thermochemical energy storage, the addition of glass fibers, and the synthesis of boron compound materials such as BN [31,32]. B_2_O_3_ is believed to enhance the electrochemical properties of MoS_2_ as it is conducted on 2D graphene materials.

In this study, we report the use of boron-oxide-nanoparticle-decorated MoS_2_ NSs as anode materials in LIBs. The MoS_2_ NSs were prepared using a chemical exfoliation method, and the decoration of B_2_O_3_ was carried out using a facile sintering technique. The results showed enhanced cycling stability in the MoS_2_ anode when B_2_O_3_ formed a crystal structure, delivering a reversible capacity of ~500 mAh∙g^−1^. These results suggest that the use of B_2_O_3_ can be a viable strategy for stabilizing anode materials for lithium storage applications.

## 2. Materials and Methods

### 2.1. Chemical Materials

Molybdenum (VI) sulfide (MoS_2_, powder, 98%), boric acid (H_3_BO_3_, powder > 99.5%), solution of n-butyllithium in hexane (2.5 M), 1-methyl-2-pyrrolidone (NMP, anhydrous, 99.5%), and polyvinylidene fluoride (PVDF, MW 534,000) were purchased from Sigma-Aldrich Inc. (St. Louis, MO, USA). Super-P amorphous carbon black (C, ~40 nm, 99.99%) was purchased from Alpha Aesar Inc. (Tewksbury, MA, USA).

### 2.2. Exfoliation of MoS_2_ NSs

The exfoliation of the MoS_2_ NSs was performed according to the method outlined in previous reports [16,33,34]. In brief, 1.0 g of MoS_2_ powder and 3 mL of butyllithium/hexane were mixed in a 10-mL vessel (placed in a glove box) to prevent the self-heating of butyllithium. The 1.6 M butyllithium/hexane was prepared by diluting the delivered 2.5 M butyllithium/hexane solution into hexane solvent. The mixture was maintained for 2 days to form Li_x_MoS_2_. Li_x_MoS_2_ was then collected via centrifugation to remove the hexane and residual butyllithium. The obtained Li_x_MoS_2_ was added to 200 mL of deionized (DI) water and placed in a sonication bath for 2 h to exfoliate MoS_2_. Finally, 1T-MoS_2_ was washed with DI water four times to remove lithium ions and then freeze-dried using a Labconco freeze dryer (Labconco Corp., Kansas, MO, USA).

### 2.3. Preparation of Boron Oxide Decorated MoS_2_ NS

For boron oxide decoration, the MoS_2_ NSs were collected after washing four times with DI water. The amount of MoS_2_ was determined by weighing the same amount of MoS_2_ NS in the solution after freeze-drying. The boric acid to MoS_2_ NS weight ratios were approximately 10, 20, and 30%. The mixtures were dispersed in DI water by sonication for 1 h, then freeze-dried, and sintered at 400 °C for 2 h in a tube furnace under Ar gas. The collected powder was denoted as MoS_2__B1, -B2, and -B3 with increasing amounts of boric acid (10, 20, and 30 wt%, respectively).

### 2.4. Material Characterization

The structure of the materials was measured by X-ray diffraction (XRD) (D/MAX-2200 Rigaku Tokyo, Japan) over the 2θ range of 10–70°. The morphologies, sizes, and detailed structures of B_2_O_3_ decorated MoS_2_ NS were analyzed using scanning electron microscopy (SEM) (Hitachi S4700, Tokyo, Japan) and transmission electron microscopy (TEM, TECNAI G2F30, FEI Corp., Hillsboro, OR, USA).

### 2.5. Electrochemical Measurements

To evaluate the electrochemical performance of the materials and their lithium storage capability, the materials were assembled as working electrodes in half-cell LIBs using a coin-type cell (CR 2032, Rotech Inc., Gwangju, Korea) with a lithium reference electrode. The active material was mixed with PVDF and carbon super P at a weight ratio of 70:15:15 in a NMP solution to form a slurry. The working electrode was prepared by casting the slurry on a copper electrode, using the doctor blading method, followed by drying in a vacuum oven at 70 °C for 24 h. The battery structures were assembled under Ar gas in a glovebox with positive pressure. The separator and electrolyte were polyethylene and 1 M LiPF_6_ in ethylene carbonate/diethylene carbonate (EC: DEC = 1:1 by volume). The galvanostatic electrochemical charge–discharge performances of the cells were measured using a battery cycle tester (WBCS3000, WonAtech, Seocho-gu, Seoul, Korea) across the voltage range of 0.01–3.0 V versus Li/Li^+^. Cyclic voltammetry (CV) tests, across a voltage range of 0.01–3.0 V, and electrochemical impedance spectroscopy (EIS), over a frequency range of 100 kHz to 0.1 Hz, were performed using ZIVE MP1 (WonAtech, Seocho-gu, Seoul, Korea). All the specific capacities were calculated based on the weights of the active materials.

## 3. Results and Discussion

**Figure 1:**
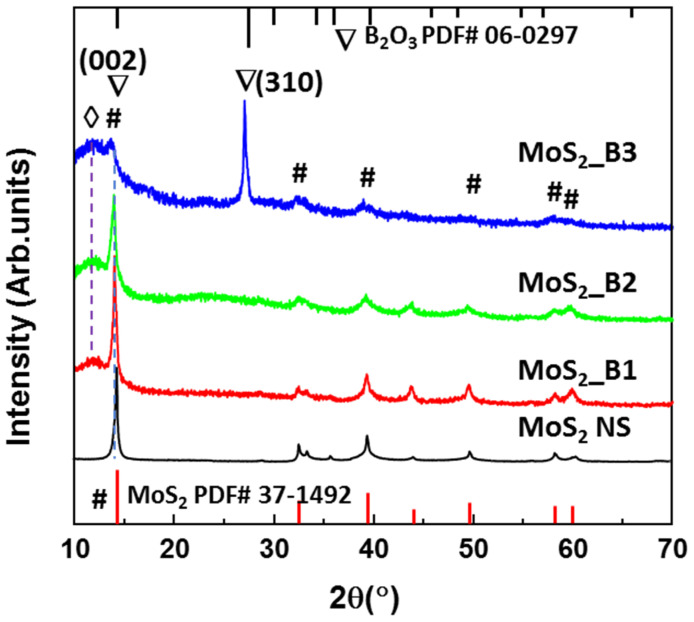
X-ray diffraction patterns of MoS_2_ NS and B_2_O_3_ decorated MoS_2__B1, -B2, and -B3 with starting H_3_BO_3_ weight percentages of 10, 20, and 30%, respectively. The symbol #, ∇, and ◊ indicate the peaks of MoS_2_ and B_2_O_3_ lattices, and stacking layer, respectively.

Figure 1 shows the XRD patterns of the MoS_2_ NSs and MoS_2__B1, -B2, and -B3 samples synthesized with 10, 20, and 30 wt% boric acid. The MoS_2_ NS exhibited a main peak at ~14.2°, indicating the main orientation of the (002) plane in the 2D structure, as per JCPDS #37-1492. The other weak peaks of MoS_2_ indicated the presence of multiple layers of these materials. These results are consistent with MoS_2_ NSs synthesized by various methods, such as hydrothermal or sonication methods [19,20,35]. The B_2_O_3_ at lower concentrations of 10% and 20% did not exhibit the peak of boric oxide, which can be due to the amorphous structures on the MoS_2_ NS surface. When increasing the boric acid to 30 wt%, the crystallinity of B_2_O_3_ was observed. The structure of B_2_O_3_ matched the cubic structure of B_2_O_3_ in JCPDS card #06-0297 with a high lattice constant (a = 10.05 Å). This lattice constant was sufficiently high compared to the 0.76 Å of lithium ion. Therefore, B_2_O_3_ coverage on MoS_2_ may not affect lithiation/delithiation. In addition, the XRD patterns of the MoS_2__B1, -B2 and B3 samples show a broad peak at ~12°. According to Bragg’s law, the lattice spacing can be calculated from the equation d=λ/2sinθ, where λ is the X-ray wavelength and θ is the diffraction angle. Therefore, the lattice spacing of this peak is ~7.37 Å, and this can be attributed to the expansion of the MoS_2_ layers or the stacking of MoS_2_ NSs with B_2_O_3_ NPs. This stacking layer had a large lithium-ion radius, thereby generating a facile path for the insertion/desertion of these ions.

**Figure 2:**
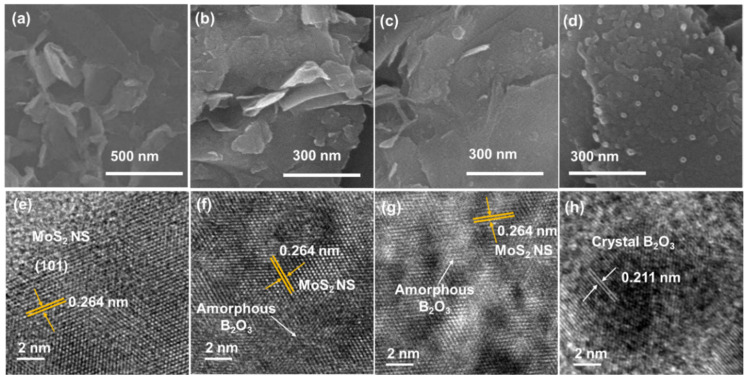
(**a**–**d**) Scanning electron microscopy and (**e**–**h**) transmission electron microscopy images of MoS_2_ NS and MoS_2__B1, -B2, and -B3, respectively.

To confirm the morphologies of the MoS_2_ NSs and their B_2_O_3_ decorations, the materials were subjected to SEM and TEM measurements, as shown in Figure 2. As seen in Figure 2a, the MoS_2_ NSs were exfoliated from the bulk material to nanosheets with a wide size ranging from 200 nm to a few micrometers. The size diversity is due to the strong reaction of intercalated lithium between MoS_2_ layers and DI water, which broke the NSs into smaller structures and the random shape of the bulk materials. This result is consistent with previous reports of MoS_2_ NSs prepared using the liquid exfoliation method [16,34,36]. Moreover, the MoS_2_ NSs with low amounts of B_2_O_3_ (10 and 20 wt% of boric acid) show a surface with tiny spots or blurred surface on the MoS_2_ NS, which are the amorphous structure B_2_O_3_ NP decorations, as illustrated in Figure 2b,c. The MoS_2__B2 sample had larger B_2_O_3_ particles on its surface. When the B_2_O_3_ increased to 30 wt%, the SEM image in Figure 2d reveals B_2_O_3_ NPs with sizes in the range of ~10–20 nm. The crystallinity of B_2_O_3_ depends on the amount of boric acid, which could be due to the large surface area of MoS_2_ NS. At low concentration, the sintering of low amount of boric acid on MoS_2_ created imperfect lattices, leading to the low crystalline structure or amorphous structure of B_2_O_3_. On the other hand, when the concentration of boric acid was high enough (>30 wt%), the complete lattices of B_2_O_3_ NPs formed, indicating the high crystalline structure of B_2_O_3_ NPs. Therefore, it is suggested that a low amount of B_2_O_3_ only forms an amorphous structure and a high amount of B_2_O_3_ (>30 wt%) is sufficient to form a crystalline structure on the surface of MoS_2_.

TEM measurements were conducted to further reveal the structure of the MoS_2_ NSs and B_2_O_3_ NPs on the MoS_2_. Figure 2e shows a high-resolution TEM (HRTEM) image of the MoS_2_ NSs. The surface image clearly shows a lattice plane spacing of approximately 0.264 nm, which corresponds to the (101) plane of MoS_2_. Thus, MoS_2_ NSs with high crystallinity were obtained. However, in the MoS_2__B1 samples, the MoS_2_ NSs were hindered by a blurred surface, which indicated the amorphous structure of B_2_O_3_, as illustrated in Figure 2f. The blurred surface area increased in MoS_2__B2 owing to the increasing amount of B_2_O_3_ amorphous structure, as shown in Figure 2g. In addition, crystalline B_2_O_3_ was observed in the MoS_2__B3 samples (Figure 2h). The lattice spacing was measured as 0.211 nm, which corresponds to the d-spacing of the B_2_O_3_ crystal. These results strongly indicated the presence of well-decorated B_2_O_3_ NPs on the MoS_2_ NS surface.

**Figure 3:**
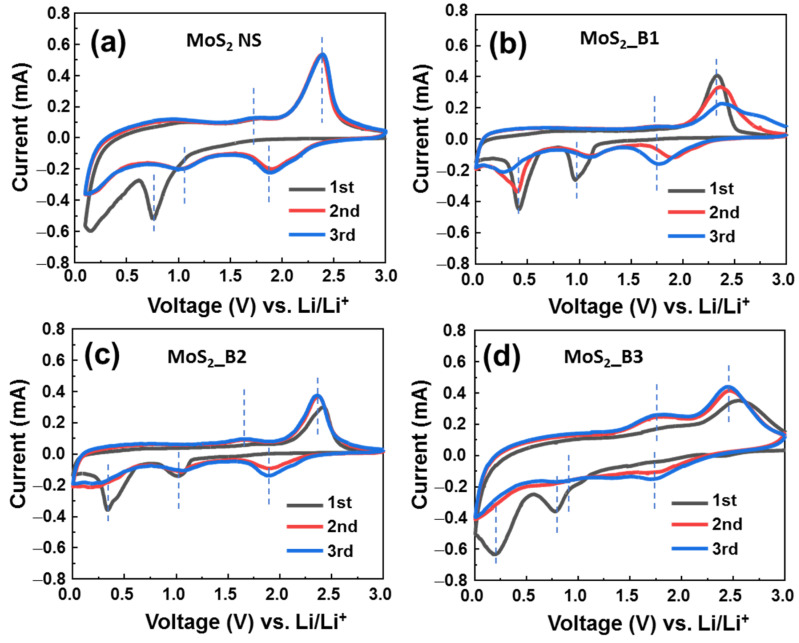
Cyclic voltammograms of (**a**) MoS_2_ NS and (**b**–**d**) of MoS_2_/B1, -B2, and -B3 electrodes, respectively.

The electrochemical properties of B_2_O_3_-decorated MoS_2_ were recorded by CV tests at a low scanning rate of 0.1 mV∙s^−1^, in the range of 0.0–3.0 V (vs. Li/Li^+^) (Figure 3). The reaction at the anode can be expressed by the following equation:

For lithiation:

(1) $${\mathrm{MoS}}_{2}+{\mathrm{xLi}}^{+}+{\mathrm{xe}}^{-}\;\to {\mathrm{Li}}_{x}{\mathrm{MoS}}_{2}$$

(2) $${\mathrm{Li}}_{x}{\mathrm{MoS}}_{2}+\left(4-x\right){\mathrm{Li}}^{+}+(4-x)e^{-}\to \mathrm{Mo}+2{\mathrm{Li}}_{2}S$$

For delithiation:

(3) $$\mathrm{Mo}\to {\mathrm{Mo}}^{4+}+4e^{–}$$

(4) $${\mathrm{Li}}_{2}S\to S+2{\mathrm{Li}}^{+}+2e^{–}$$

Finally, the solid electrolyte reaction at first cycles:

(5) $${\mathrm{Li}}^{+}+e^{-}+\mathrm{electrolyte}\to \mathrm{SEI}$$

As shown in Figure 3a, the bare MoS_2_ materials show a cathodic peak in the first cycle at ~0.76 V, which is the lithiation process to form Li_x_MoS_2_ and the deep lithiation to form Mo and Li_2_S, as shown in Equations (1) and (2). The peak between 0.1–0.5 V could be due to the formation of the SEI layer (5). These results are consistent with previous reports on 1T MoS_2_ in the first CV cycle [15,16]. From the second cycle, redox couple peaks were recorded at 1.05/1.72 V and 1.87/2.38 V, which are the reactions in Equations (2) and (3); and Equations (1) and (4), respectively. The CV curves of anodes MoS_2__B1 and B2 were similar. In these two anodes, the first cycle shows cathodic peaks at ~0.96 and 0.41 V, which correspond to the reactions (1) and (2), respectively. The anodic peaks were located at ~1.7 and 2.3 V, which correspond to the reactions (3) and (4), respectively. SEI layer formation was recorded together with the peak of reaction (2) at ~0.41 V. It is noted that the bare MoS_2_ NSs and MoS_2__B1 and -B2 electrodes show the strong redox couple peaks at 1.87/2.38 V vs. Li^+^/Li (corresponding to (1) and (4) reactions) and the weak redox couple peaks at ~1.05/1.72 V vs. Li^+^/Li (corresponding to (2) and (3) reactions). This emphasizes the hard oxidation of Mo to Mo^4+^, thus leading to the degradation of the cycling stability. In contrast, for MoS_2__B3 electrode, the formation of B_2_O_3_ crystals was significantly effective in improving the electrochemical properties of the MoS_2_ NSs. In the first cycle, the cathodic scan showed two peaks at 0.78 and 0.20 V vs. Li^+^/Li, which corresponds to the lithium insertion into MoS_2_ (Equation (1)) and the deep insertion of Li into MoS_2_/formation of SEI layer (Equations (2) and (5)). The peaks of the MoS_2__B3 anode were positioned at lower potential compared to those of MoS_2__B1 and -B2 electrodes, which were at ~0.9 and 0.4 V vs. Li^+^/Li. This peak shift could be due to the formation of B_2_O_3_ crystalline introducing a different interface to the electrolyte in comparison to the amorphous B_2_O_3_, which leads to the harder diffusion of Li in the first cycle. From the second cycle, the redox couple peaks were recorded at 0.82/1.73 V and 1.77/2.42 V, which correspond to the reactions (2) and (3); and Equations (1) and (4), respectively. The third cycle showed a similar curve to the second cycle, indicating the stable electrochemical reaction after the first cycle. Furthermore, the relative intensity of Mo’s oxidation peak located at ~1.73 V for the MoS_2__B3 anode (Equation (3)) was significantly enhanced in comparison to those of MoS_2__B1 and -B2 and bare MoS_2_ NSs anodes. It is noted that the insertion of Li in MoS_2_ at high potential is relative to the formation of a gel-like polymeric SEI layer due to the S dissolution in electrolyte [37]. The MoS_2__B1, -B2, and bare MoS_2_ NSs anodes show a high cathodic peak at ~1.87 V after three cycles, which is higher than that located at 1.77 V of MoS_2__B3 anode, indicating the higher amount of S was dissolved in electrolyte. Therefore, the MoS_2__B3 anode has high amount of recovered MoS_2_ NS, resulting in the high oxidation peak intensity of Mo to Mo^4+^. This could be due to the stability of crystalline B_2_O_3_ allowing the insert/desertion of Li ions [38]. Moreover, the sulfur atoms have high electron affinity, thus, they could not pass through the B_2_O_3_ lattice [39]. It indicates that the crystalline B_2_O_3_ effectively protected the MoS_2_ layer, preventing the loss of S atoms and the formation of gel-like polymeric SEI layer.

**Figure 4:**
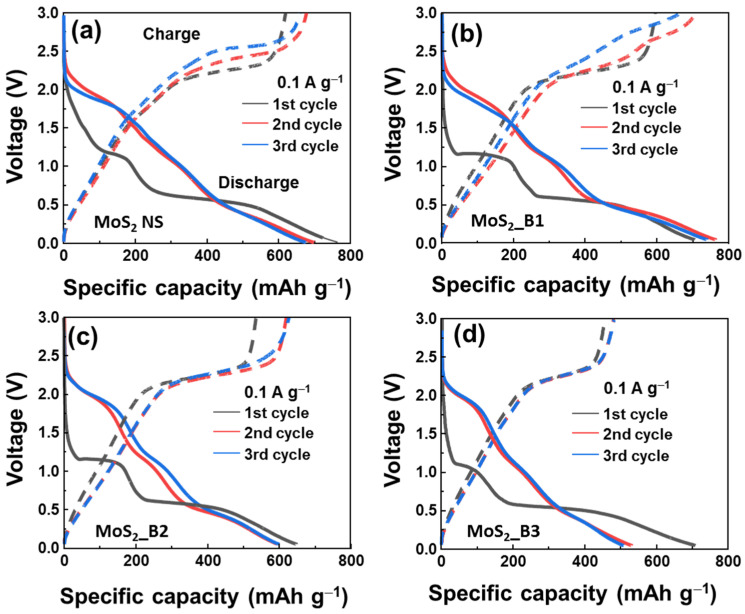
Initial voltage profiles of (**a**) MoS_2_ NS and (**b**–**d**) MoS_2__B1, -B2, and -B3 electrodes, respectively.

To further observe the effect of B_2_O_3_ on the MoS_2_ materials, the initial voltage profiles of B_2_O_3_ decorated samples are shown in Figure 4. The first three cycles of MoS_2__B1, -B2, and MoS_2_ NSs seem to be unstable, showing a clear change from the first to the second and third cycles. The voltage plateau of the first discharge curve was slightly reduced from the MoS_2_ NSs to the MoS_2__B1, -B2, and -B3 samples, where two plateaus at 1.1/0.51 to 1.1/0.51, 1.1/0.50, and 1.1/0.48, respectively, are shown. This indicates that the B_2_O_3_ crystals in the MoS_2__B3 samples changed the lithium insertion potential. In the second and third cycles, the voltage plateaus were similar for the MoS_2__B3 electrode, thereby indicating stable electrochemical properties from the second cycle. In addition, the initial discharge capacity of these anodes was high, but it reduced after each cycle owing to the formation of the SEI layer and degradation behavior in lithium ion batteries, such as cracks, sulfur dispersion in the electrolyte, and dendrite growth [40]. The initial discharge capacities for the bare MoS_2_ NSs and MoS_2__B1, -B2, and -B3 were 747.1, 717.7, 638.1, and 717.2 mAh∙g^−^^1^, respectively. The difference in the initial discharge capacities also depended on the formation of the SEI layer and the binding of B_2_O_3_ to MoS_2_. The B_2_O_3_ was reported as a low lithium ion storage capability [41]. Therefore, the increased amount of B_2_O_3_ in MoS_2_ led to the decreased charge/discharge capacities of MoS_2_ anode materials. In particular, the charge/discharge capacities of the MoS_2__B3 anode in the third cycle were 505.2/475.0 mAh∙g^−1^. 

**Figure 5:**
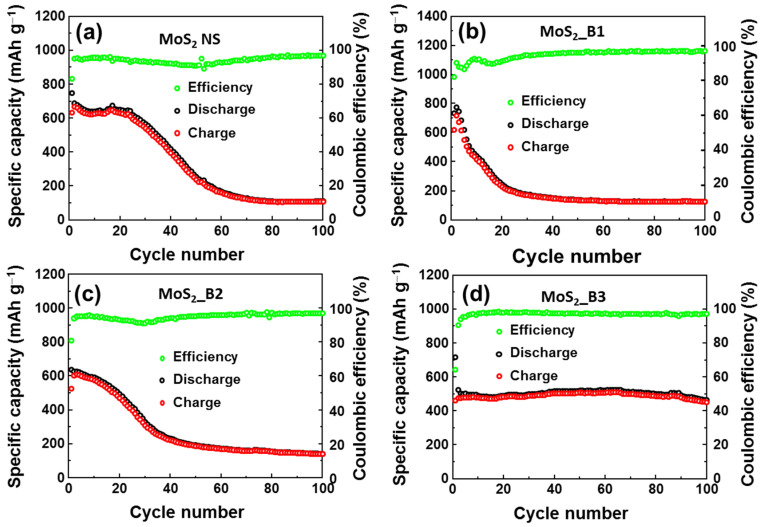
Cyclic performance of (**a**) MoS_2_ NS and (**b**–**d**) MoS_2__B1, -B2, and -B3 electrodes, respectively.

To evaluate the stability of the anode materials, cycling tests were performed at a current rate of 0.1 A∙g^−1^ for 100 cycles, as illustrated in Figure 5a–d. Detailed comparison of specific capacities of as-prepared anode materials are also shown in Table 1. The MoS_2_ NSs showed stability for ~20 cycles, and its capacity was subsequently dramatically reduced and maintained at only ~109 mAh∙g^−1^ at the 100th cycle (Figure 5a). The addition of B_2_O_3_ also resulted in very fast degradation, and the remaining capacity was ~125 mAh∙g^−1^ and ~140 mAh∙g^−1^ at the 100^th^ cycle in the MoS_2__B1 and B2 anodes, respectively (Figure 5b,c). The MoS_2__B1 and B2 showed the enhancement of lattice spacing of MoS_2_, facilitating the insertion/desertion of Li ions. However, the amorphous B_2_O_3_ could not prevent the loss of S atoms. Therefore, MoS_2__B1 and B2 anodes exhibited inferior stability to the pure MoS_2_ NS. In contrast, the crystalline B_2_O_3_ in the MoS_2__B3 electrode showed a high capacity in the first cycle, and it demonstrated prolonged cycling stability for 100 cycles. As shown in Figure 5d, the capacity of MoS_2__B3 at the 100th cycle was ~451 mAh∙g^−1^, which was 86.2% of the second cycle (~510 mAh∙g^−1^) and ~62.9% of the first cycle. Therefore, it can be concluded that the enhancement of the redox reaction with the B_2_O_3_ crystals was effective in improving the stability of the MoS_2_ NSs.

**Figure 6:**
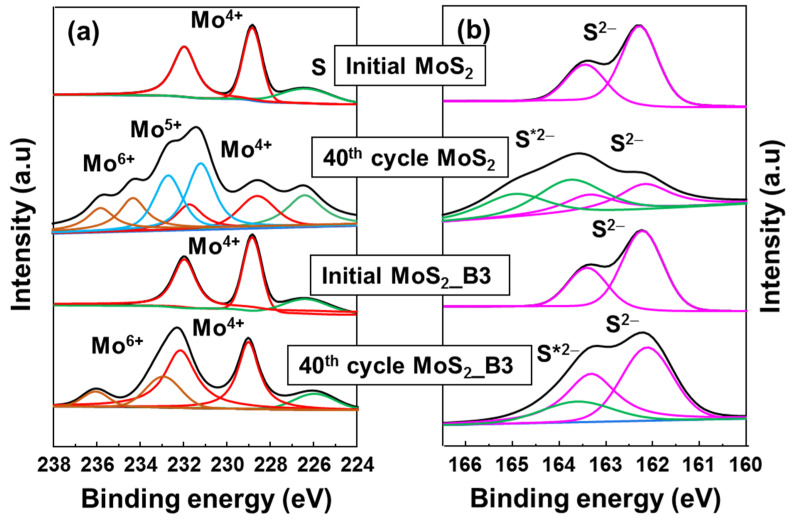
Ex-situ high resolution XPS spectra of (**a**) Mo 4f and (**b**) S 2p for MoS2 NS and MoS2_B3 anodes at initial state and after cycling for 40 cycles. The symbol * indicates the shifted S 2p peaks.

The protective role of B_2_O_3_ was further confirmed via ex-situ XPS spectra, as shown in Figure 6. Both bare MoS_2_ NS and MoS_2__B3 anodes were compared at the initial state and at 3.0 V 40 cycles. The initial state of MoS_2_ and B_2_O_3_ decorated samples presented the same conditions of Mo^4+^ and S^2−^. However, after 40 cycles, bare MoS_2_ NSs showed significant change in Mo 4f peak. Mo^4+^ peak intensity reduced, and the Mo^5+^ and Mo^6+^ peaks appeared. Moreover, in S 2p spectrum, the S 2p peak split to S^2−^ peak at ~162 eV and a S*^2−^ peak at ~163.6 eV, which might be related to the formation of polymeric SEI layer due to the S dissolution or the unrecoverable Li_2_S [42,43], indicating unstable MoS_2_ NS anode. On the other hand, the MoS_2__B3 anode show a better stability, where the main peak of Mo 4f assigned to Mo4+ was maintained with partial Mo^6+^ peaks. It is noted that the Mo^6+^ peak might appear due to the sample preparation method as pointed out in previous reports [44,45,46]. The S 2p peak of MoS_2__B3 showed a small change with S*^2−^ peak, which might be due to a partial loss of S to polymeric layer or unrecoverable Li_2_S. These results indicate that B_2_O_3_ layer efficiently protected MoS_2_ layer, preventing the loss of S to electrolyte. The small amount of S loss can be further improved after optimizing the B_2_O_3_ layers.

**Figure 7:**
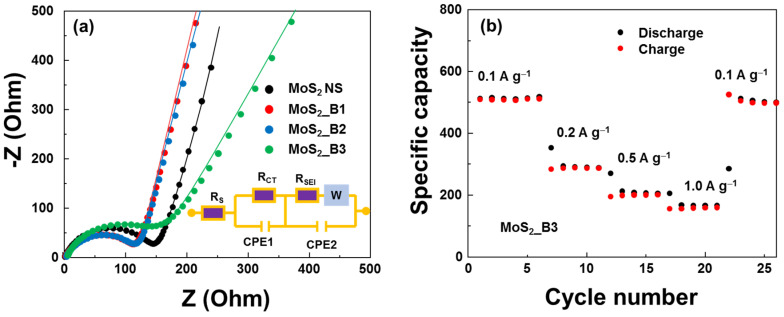
(**a**) Nyquist plots of MoS_2_ and MoS_2__B1, -B2, and -B3 anodes; and (**b**) rate performance of MoS_2__B3 anodes.

The electrical properties of the anode materials were evaluated using EIS measurements, as shown in Figure 7a. The equivalent circuit (modified Randle’s model) contained series resistance (R_S_), charge-transfer resistance (R_CT_), SEI layer resistance (R_SEI_), a diffusion Warburg impedance element, and constant phase elements (CPE1 and CPE2). The extracted R_CT_s of the MoS_2_ NS and MoS_2__B1, -B2, and -B3 samples were 150.3, 118.4, 118.2, and 148.6 Ω, respectively. The addition of boron oxide did not significantly affect the resistance of the anode material. MoS_2__B1 and -B2 showed reduced resistance. Then, the resistance increased in MoS_2__B3 when B_2_O_3_ formed crystallinity owing to the low conductivity of B_2_O_3_. However, this resistance was still lower than that of the bare MoS_2_. As a 2D layered structure material, the conductivity of MoS_2_ decreases when the number of layers is reduced. In addition, the presence of B_2_O_3_ NPs may prevent the restacking of MoS_2_ NSs and the NS material from forming a bulk structure, thereby enhancing the conductivity of the anode material. The rate performance of MoS_2__B3 is shown in Figure 7b. An increase in the current rate led to a decrease in capacity. At 1.0 A∙g^−1^, the capacity was maintained at ~155 mAh∙g^−1^. Nevertheless, the MoS_2__B3 electrode can be recovered to almost 98% when decreasing the current rate to 0.1 A∙g^−1^, thus illustrating a highly reversible rate performance. 

The recent works on the modification of MoS_2_ are shown in Table 2. The initial discharge capacities were high above 800 or even 1200 or 1400 mAh∙g^−1^. This might be due to the contribution to the capacity of the modified materials. From our method, B_2_O_3_ does not mainly contribute to the capacity, but effectively protects the MoS_2_ layer, which maintains the highly stable capacity. Moreover, the sintering method and the utilization of the boric acid are cost-effective ways. Therefore, it can be readily scale-up to the industrial size. We also believe that the use of carbon-based and co-active materials can further improve the electrochemical performance of the materials presented in this study for lithium ion storage application.

## 4. Conclusions

In summary, B_2_O_3_ NP-decorated MoS_2_ NSs were successfully fabricated via a facile chemical exfoliation and sintering process. The XRD, SEM, and TEM measurements confirmed that the crystalline B_2_O_3_ could be formed at high boric acid content of over 30 wt%. The presence of B_2_O_3_ created the lattice spacing of ~7.37 Å in MoS_2_ NS. Crystal B_2_O_3_ formed with a lattice spacing of ~2.58 Å, improving the redox reaction in the conversion of MoS_2_ during the cycling process. The high intensity of Mo oxidation peak and the lower potential of lithium insertion into MoS_2_ indicated B_2_O_3_ layer played a role as a protective layer, preventing the dissolution of S atoms into electrolyte. The bare MoS2 material and amorphous B_2_O_3_ in MoS_2__B1 and -B2 anodes showed fast degradation after 20–40 cycles due to the loss of sulfur into the electrolyte. Meanwhile, the MoS_2__B3 electrode with protectable crystalline B_2_O_3_ layer demonstrated a stable capacity of ~500 mAh∙g^−1^ and a high-capacity retention of ~86.2% after 100 cycles. These results suggest that B_2_O_3_ NP decorations on anode materials could be a potential approach for high-stability anodes in lithium storage applications.

## Figures and Tables

**Table 1:** Comparison of specific capacities of bare MoS_2_ NS and B_2_O_3_ decorated MoS_2_ anodes.

Anode	Initial Capacity (mAh g^−1^)	Current Rate (A∙g^−1^)	Capacity after 100 Cycles (mAh∙g^−1^)
Bare MoS_2_	747.1	0.1	109
MoS_2__B1	717.7	0.1	125
MoS_2__B2	638.1	0.1	140
MoS_2__B3	717.2	0.1	451

**Table 2:** Comparison of the electrochemical performance of the modified MoS_2_ anodes in lithium-ion batteries.

Anode Material	Current Density (A∙g^−1^)	Initial Discharge Capacity (mAh∙g^−1^)	Cycle Number	Specific Capacity (mAh∙g^−1^)	References
Ag decorated MoS_2_	0.1	~900	100	510	[16]
TiO_2_ decorated MoS_2_	0.1	827	100	604	[17]
SiCN-MoS_2_	~0.1	~726	20	445.6	[47]
Carbon coated MoS_2_	0.1	1419	50	837	[48]
MoS_2_ on CNT	0.025	1200	50	650	[49]
B_2_O_3_ on MoS_2_	0.1	717	100	451	This work

## Data Availability

The data presented in this study are available on request from the corresponding author.
